# Draft genome sequence of *Cupriavidus necator* DVZ60, an isolate from iodine-impacted Hanford groundwater

**DOI:** 10.1128/mra.00449-26

**Published:** 2026-07-17

**Authors:** Oluwatomiwa J. Sunbare-Funto, Raissa K. Kodia-Batamio, Jerome J. O. Oliver, Charnae A. Henry-Smith, Danielle L. Saunders, M. Hope Howard, Brady D. Lee, Courtney J. Robinson

**Affiliations:** 1 Department of Biology, Howard University 8369, Washington, DC, USA; 2 Pacific Northwest National Laboratory, Energy and Environment Directorate 6865, Richland, Washington, USA; 3 Savannah River National Laboratory, Environmental and Legacy Management Directorate 1073, Aiken, South Carolina, USA; University of Maryland School of Medicine, Baltimore, Maryland, USA

**Keywords:** *Cupriavidus *

## Abstract

*Cupriavidus necator* DVZ60 was isolated from a sediment trap deployed in a ^129^I groundwater plume at the Hanford Site (Washington, USA). Genome sequencing of the strain led to the generation of 84 contigs and indicated that the genome is 7.552 Mb in size, with 66.2% GC content.

## ANNOUNCEMENT

From 1943 to 1987, Hanford Site operations discharged approximately 1 billion m^3^ of liquid wastes, including radioactive constituents that affected local ecosystems (1). ^129^I, a byproduct of plutonium enrichment, is a mobile, potentially toxic, and long-lived radionuclide. Culturing bacteria from groundwater at the Hanford Site using a sediment trap incubated for 50 days in a ^129^I plume, where ^129^I concentrations average ~3.5 pCi/L and iodate is the dominant iodine species, resulted in multiple isolates. One of the isolates was *Cupriavidus necator* DVZ60, which was identified originally by 16S rRNA gene sequencing (2). Draft genomes of other isolates from the ^129^I plume have been reported, including those of *Pseudomonas* strains DVZ6 and DVZ24 (3). Here, we describe the draft genome of *C. necator* DVZ60 with the goal of eventually understanding the genetic basis underlying biological drivers of iodine transformation at the Hanford Site and the genetic capacity of *C. necator* as a species.

*C. necator* DVZ60 was grown from an −80°C freezer stock in tryptic soy broth by shaking at 28°C for 48 h, and genomic DNA was purified with the Invitrogen PureLink Genomic DNA Mini kit. Before whole-genome sequencing, the identity of the isolate was confirmed by colony PCR targeting the nearly full-length 16S rRNA gene, followed by Sanger sequencing of the amplicon and BLAST comparison to reference sequences in GenBank, which supported its designation as *C. necator* (top 16S rRNA gene hit, NR_102851.1, 99% query coverage, and 99.93% identity).

Genome sequencing was completed at SeqCenter in Pittsburgh (https://www.seqcenter.com/). Library samples were prepared using an Illumina DNA Prep kit and sequenced on an Illumina NextSeq 2000 instrument with 10 bp unique dual indexes to generate 2 × 151 bp paired-end reads. Adapters were demultiplexed, checked for quality, and trimmed using bcl-convert v3.9.3. *De novo* assembly of reads was done using Unicycler (v0.5.1) (4), SPAdes (4.2.0) (5), and MEGAHIT (v1.2.9) (6). Based on QUAST (v5.3.0) metrics, MEGAHIT produced the best assembly and was chosen for downstream analyses (7). Genomic completeness and contamination were estimated with CheckM2 (v1.0.0) (8) and BUSCO (v.6.0.0) (9). The NCBI Prokaryotic Genome Annotation Pipeline (v6.10) was used to annotate the genome (10). The digital DNA-DNA hybridization analysis and species identification of the genome were also performed at the Type (Strain) Genome Server (https://tygs.dsmz.de) (11).

The genome size of *C. necator* DVZ60 is 7,552,451 bp, the G + C content is 66.2%, with 42 tRNAs, and 5 rRNAs. CheckM2 estimated 100% completeness with 1.9% contamination, and BUSCO reported >99% completeness. Other assembly and sequencing data can be found in Table 1. The species designation was confirmed with a dDDH (formula d4) value of 94.6% (95% CI, 93.0–95.9) when compared to the genome of *C. necator* Makkar and Casida 1987 (NBRC 102504; formerly known as *Wautersia eutropha*), and an ANI >99% when compared to *C. necator* PHE3-6 (NBRC 110655). This study also revealed the presence of genes predicted to encode multicopper oxidases, peroxidases, and reductases, which have been implicated in iodine biotransformation and could be targets for future investigations (12).

**TABLE 1:** Assembly and annotation summary of *C. necator* DVZ60

Parameters	Genome data
Bioproject	PRJNA855226
BioSample	SAMN53300215
Sequence Read Archive accession	SRR36125207
Genome assembly accession	GCA_053747635.1
Total number of reads	9,594,805
Total read length (bp)	2,465,078,123
Avg. read length (bp)	128
Genome coverage (×)	325.8
Total assembly size (bp)	7,552,451
Number of contigs (≥1 kb)	84
*N*_50_ (bp)	194,368
*L*_50_	12
Longest contig (bp)	656,006
GC content (%)	66.2
Number of coding sequences	7,191
rRNA	5
tRNA	42

## Data Availability

The Whole Genome Shotgun assembly has been submitted to GenBank accession number JBSMJC000000000; the version used in this report is JBSMJC000000000.1. Raw Illumina reads are deposited in the Sequence Read Archive as SRR36125207.
